# Implementation of Rapid Drug Desensitization in Antineoplastic Drug Therapy in Denmark Using One‐Bag Protocols

**DOI:** 10.1002/clt2.70093

**Published:** 2025-08-13

**Authors:** Trine Holm Rasmussen, Charlotte Gotthard Mortz, Per Pfeiffer, Nina Andersen, David George Mawn, Line Kring Tannert, Millie Nguyen Basu, Helene Marlies Rasmussen, Carsten Bindslev‐Jensen

**Affiliations:** ^1^ Department of Dermatology and Allergy Center Odense Research Center for Anaphylaxis (ORCA) Odense University Hospital Odense Denmark; ^2^ Department of Oncology Odense University Hospital Odense Denmark; ^3^ Department of Hematology Odense University Hospital Odense Denmark; ^4^ Hospital Pharmacy Funen Cytostatic Production Odense University Hospital Odense Denmark

**Keywords:** cancer, chemotherapy, desensitization, drug allergy, hypersensitivity

## Abstract

**Background:**

Rapid drug desensitization (RDD) is the cornerstone of managing patients with immediate drug hypersensitivity reactions (IDHR) to antineoplastic drugs in Southern Europe and the United States. As the first in Northern Europe, an allergy treatment program that includes RDD and drug provocation testing (DPT) was implemented for Danish patients with cancer. We report the results of this allergy intervention, the number of successful treatments, the fraction, timing and severity of breakthrough reactions (BTR) and the actual treatment duration of RDD procedures.

**Methods:**

This was a prospective observational study. Patients with IDHRs to antineoplastic drugs referred to the allergy treatment program were included. Patients were followed up until finalization of DPT and/or RDD. RDDs were performed according to one‐bag RDD‐protocols with drug concentrations strictly following manufacturer's instructions and infusion sets primed with flushing fluid. The outcome of DPTs and RDDs were recorded together with detailed information on BTRs and treatment duration of RDD‐procedures.

**Results:**

During 28 months, 72 patients were included. With DPT, a safe drug alternative was found for five drugs, hypersensitivity was ruled out for six, and one treatment was discontinued after a positive DPT. RDD was performed in 60 patients. Of 248 initiated RDD procedures, 247 were completed. BTRs were observed in 53% of patients and 27% of RDD‐procedures, with most BTRs being mild to moderate. The treatment duration was below 6 hours in 96% of RDD procedures.

**Conclusion:**

The allergy treatment program, which included DPT and one‐bag RDD‐protocols, allowed patients to continue critical antineoplastic treatments despite IDHRs.

**Abbreviations**
BTR: Breakthrough reaction
DPT: Drug provocation test
EAACI: European Academy of Allergy and Clinical Immunology
IDHR: Immediate drug hypersensitivity reaction
OUH: Odense University Hospital
RDD: Rapid drug desensitization
SCAR: Severe cutaneous adverse reaction
SPT: Skin prick test

## 1 Introduction

Antineoplastic treatments improve survival and quality of life for patients with cancer. Immediate drug hypersensitivity reactions (IDHR) can prevent patients from receiving critical treatments. In a Danish population, 1.1% of patients undergoing antineoplastic drug therapy discontinue treatment due to IDHRs [1]. Therefore, we introduced drug provocation testing (DPT) and rapid drug desensitization (RDD) as treatment options for these patients.

Ideally, RDD should be performed in patients with confirmed hypersensitivity [2, 3]. Skin testing with antineoplastic drugs is used for risk assessment prior to RDD [3, 4, 5, 6, 7, 8]. Skin testing with chemotherapeutics must be performed in laminar flow chambers to ensure staff safety and avoid environmental exposure [9]. Some centers use DPT to confirm or rule out hypersensitivity in selected patients [3, 10, 11, 12].

The most validated RDD‐protocols have incorporated data from in vitro models of mast cells undergoing RDD [2, 13, 14]. RDD‐protocols involve two to four infusion bags (standard is three bags) with initial infusion rates ranging from 1:10,000 to 1:100 of the standard rate [5, 6, 12, 15, 16, 17]. The infusion rate is doubled every 15 min until reaching the standard infusion rate. The estimated treatment duration ranges from 4 to 16 h (standard is six hours [6]). The actual treatment duration, including time to change infusion bags and to treat any complicating reactions, has not been reported. Limitations of multi‐bag RDD‐protocols include compromising recommended drug concentrations, increased workload for the pharmacy and risk of human errors, as infusion bags are often not fully infused.

One‐bag RDD‐protocols with a similar efficacy to three‐bag RDD‐protocols have emerged in recent years [18, 19, 20, 21, 22, 23] and been shown to reduce treatment duration [19, 22]. The challenge is that initial infusion rates are as low as 0.1 mL/h. Infusion sets are primed with antineoplastic drug solution instead of flushing fluid (saline or glucose) in order to accurately deliver the initial tiny drug doses. This means that antineoplastic drugs are not contained within closed systems, which can result in environmental exposure.

The use of premedication is controversial. Some centers provide standard premedication as recommended by the manufacturer [2, 10, 12]. Others routinely provide additional premedication, including antihistamines, montelukast, ASA/NSAID, steroids and benzodiazepines [6, 8, 15, 24]. The actual use of premedication has not been previously published, nor has the use of rescue medication for treatment of BTRs.

In Southern Europe and the United States, RDD is a cornerstone in the management of patients experiencing IDHRs to antineoplastic drugs. RDD allows close to 100% of patients to continue treatment with offending drugs [2, 3]. Breakthrough reactions (BTR) (IDHRs occurring during RDDs) are often mild to moderate in severity. They occur in 39%–42% of patients and 27%–42% of RDD procedures [2]. Despite promising results, RDD is not offered to patients reacting to antineoplastic drugs in Northern Europe.

Using one‐bag RDD‐protocols with drug concentrations following manufacturer's instruction and infusion sets primed with flushing fluid, the objectives of this study were to investigate: (I) the proportion of patients able to continue treatment with culprit drugs via DPT and/or RDD, (II) the proportion and severity of BTRs, (III) the timing of BTRs, (IV) the use of premedication and rescue medication, and (V) the duration of RDD procedures and antineoplastic treatments.

## 2 Methods

### 2.1 Study Design and Participants

An allergy treatment program including DPT and RDD was implemented at the Allergy Center at Odense University Hospital (OUH), Denmark, in close collaboration with the Departments of Oncology and Hematology, OUH. The study is a prospective observational study of adult patients referred to the allergy treatment program between 01.08.2022 and 30.11.2024. Patients were followed up until finalization of DPT and/or RDD.

An IDHR was defined as signs and symptoms of hypersensitivity that occurred during or within 6 hours after treatment. Patients with IDHRs to antineoplastic drugs could be referred to the allergy treatment program. There were no requirements regarding severity of IDHRs or re‐treatment attempts. The treating oncologist/hematologist determined the indication for referral. Allergists first became involved in the treatment of patients when patients were referred to the allergy treatment program.

### 2.2 Allergy Work‐Up

An allergy work‐up was performed before next scheduled treatment, within few days and up to 3 weeks after the index‐IDHR (index‐IDHRs are IDHRs that occurred at cancer departments). Information about index‐IDHRs was obtained from the patient and from medical records. This included potential culprit drugs, timing of subjective and objective symptoms, and measured vital signs. IDHRs were categorized by severity and reaction phenotype based on clinical history as suggested by recognized research groups, see Table S1 [12, 25]. The severity was graded according to the RCUH‐classification [12]: grade 1: mild, grade 2: moderate, grade 3: severe, grade 4: anaphylactic shock. The reactions phenotypes were: *type‐1*, *cytokine‐release*, *mixed‐type* and *either‐type* [25]. Type‐1 reactions were considered an indicator of a possible IgE‐mediated mechanism. An IgE‐mediated mechanism could also be suspected in other reaction phenotypes if patients reacted to drugs previously tolerated [25, 26, 27].

Skin testing for biologics (checkpoint inhibitors and *other* biologics) was performed in duplicate: skin prick testing (SPT) in undiluted concentration and intradermal testing in a 1:100 concentration, two to 3 weeks after the index‐IDHR. Skin testing was not performed with chemotherapeutics for legal reasons. SPT was performed to relevant drug excipients as previously described [28]. A 10 mg/mL histamine solution and saline were used as positive and negative controls, respectively. Wheals 3 mm larger than the negative control were considered positive.

An imputability analysis was performed based on clinical history and skin test results (when available) to determine the probability that symptoms observed during the index‐IDHR were caused by the suspected culprit drug [29]. Next treatment was performed as a DPT or RDD, depending on the results of the imputability analysis and a risk assessment (Supporting Information S1). DPT was selected if symptoms observed during the index‐IDHR could not be reliably attributed to the suspected culprit drug. DPT was deselected if the index‐IDHR was an anaphylactic reaction or a presumed IgE‐mediated reaction (type‐1 phenotype), or if re‐treatment had already been attempted and failed in cancer departments. Treatment with alternative drugs that involved a risk of cross‐reactivity was tested in DPTs.

### 2.3 Timing, Location and Standard Precautions

DPT and RDD were performed in line with next planned treatment. Antineoplastic treatments were prescribed by the referring oncology/hematology department and administered at the Allergy Center. Rescue medication and resuscitation equipment were immediately available.

Allergy nurses performed DPT and RDD procedures under supervision of an allergist. Patients were evaluated before and after treatment, and by indication, using objective measurements, including lung function tests, blood oxygen saturation, blood pressure, heart rate, and temperature.

An oncology/hematology nurse assessed patients before initiating treatments and ensured proper handling of antineoplastic drugs.

### 2.4 Drug Provocation Testing

DPTs were carried out according to EAACI guidelines [3]. Culprit drugs were administered according to standard procedures. A graded DPT could precede a standard DPT. A restart protocol was used in case of an IDHR during DPT to ensure the patient received a complete treatment.

### 2.5 Rapid Drug Desensitization

One‐bag RDD‐protocols were used, with drug concentrations following manufacturer's instruction. The exception was bendamustine, where two infusion bags were used due to its short shelf life. The volumes of infusion bags were: 60, 110, 265, 525, and 1025 mL. See Supporting Information S2 for information on drug concentrations and carrier fluids. Infusion sets were primed with 25–27.5 mL flushing fluid. Encoded programs on high‐precision infusion pumps (Infusomat Space Braun) ensured precise delivery of drug doses. The venous access was a peripheral venous catheter or a *port‐a‐cath*, both with a Y‐port. Antineoplastic drugs were administered in one port and saline/glucose at a flow of 150 mL/h in the other.

The original standard one‐bag RDD‐protocol included a 20‐mL flush step infused over 15 min to remove flushing fluid. The initial infusion rate was 1:800 of the standard rate. The rate was doubled every 15 min until reaching a final rate of 50% (Table S4).

The standard infusion duration was 1 hour for platinum salts, paclitaxel 80 mg/m^2^ (but 3 hours for paclitaxel 175 mg/m^2^), docetaxel, liposomal doxorubicin, bendamustine, etoposide, checkpoint inhibitors and *other* biologics.

**FIGURE 1:**
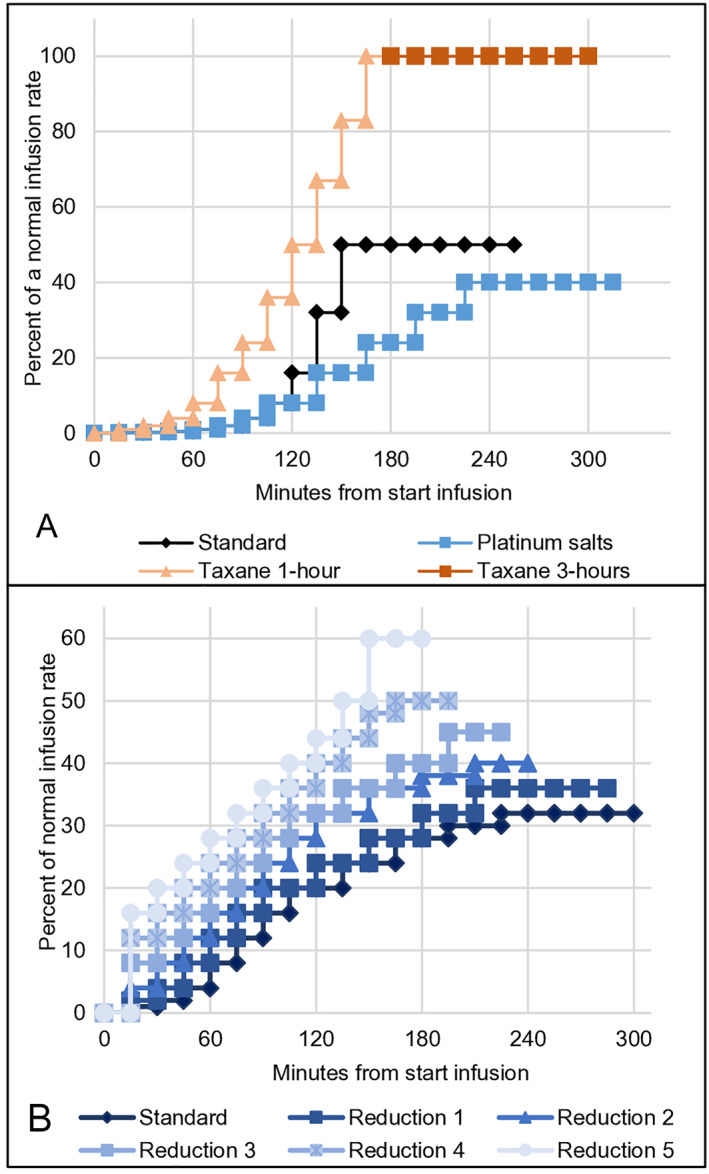
Examples of adjusted one‐bag RDD‐protocols for different drug classes. (A) Adjusted standard one‐bag RDD‐protocols for platinum salts and taxanes in relation to the original standard protocol. During the flush step, 16 and 20 mL are infused over 15 min for taxanes and platinum salts respectively. Depicted are percentages of a standard infusion rate at different time points. Dose increases for taxane 1‐h and taxane 3‐h regimens follow the same protocol. The duration of the infusion is 2 h longer for taxane 3‐h as the drug dose is higher. This is in accordance with the standard guidelines followed in the oncology department. (B) Adjusted standard 1‐bag RDD‐protocol for checkpoint inhibitors and 5 consecutive standardized reduction plans. Depicted are percentages of a normal infusion rate at different time points.

In January 2024, the standard one‐bag RDD‐protocol was customized for the drug classes: platinum salts, taxane 1‐h, taxane 3‐h, liposomal doxorubicin, checkpoint inhibitors and *other* biologics. Adjustments were made based on observations of timing, severity, and phenotype of BTRs observed in different drug classes. For example, the flush step was lowered to 16 mL for taxanes and the final infusion rate was increased to 100%, whereas dose escalations in the final steps for platinum salts were reduced (Figure 1A and Table S5). If a culprit drug exhibited an atypical reaction phenotype for its drug class, a more appropriate infusion plan could be selected based on that phenotype.

Infusion duration was incrementally shortened after uneventful RDDs. Five consecutive standardized reduction‐plans were defined for different drug classes (Figure 1B provides an example). Patients continued to standard treatment if *reduction‐plan‐5* was tolerated. This was relevant for patients receiving more than six treatments (e.g. patient treated with weekly paclitaxel or checkpoint inhibitors).

When BTRs occurred, the infusion was paused and medical treatment applied if indicated. Infusion was resumed without altering the infusion plan when symptoms subsided. Adjustments to infusion plans or premedication for the next planned treatment were made based on severity and phenotype of BTRs. However, infusion plans could remain unchanged following easily controllable mild to moderate BTRs.

Timing, symptoms, and severity of BTRs were recorded, along with usage of rescue medication. When BTRs occurred, the volume infused (including flushing fluid) was recorded in milliliters and as a fraction of the target dose.

Duration of RDD procedures included initial and final flush steps, and any delays due to BTRs. The treatment duration included the RDD procedure and time spent to administer additional antineoplastic drugs. Time measurement did not include initial and post‐treatment assessment and observation time.

### 2.6 Premedication

Premedication was administered according to manufacturer's instructions. The only exception was that antihistamines were avoided. Glucocorticoids were reduced or discontinued after uneventful RDDs, unless they were used to prevent nausea. Premedication could be added after RDDs involving BTRs: glucocorticoids to prevent fever and vomiting, paracetamol to prevent fever and back pain, and omalizumab was considered after severe *type‐1* phenotype BTRs that occurred at low doses. The usage of premedication was recorded.

### 2.7 Statistical Analysis

Descriptive statistics was performed. Results are given as median, range and proportions (%), using StataNow 18 BE (StataCorp, College Station, TX, USA).

### 2.8 Ethics

This study was approved by the Ethics Committee of Southern Denmark (project‐ID S‐20220004), and the Danish Data Protection Agency (Journal 22/9106). Written informed consent was obtained from all participants.

## 3 Results

### 3.1 Patient Characteristics

During a 28‐month period, 72 patients were referred to the allergy treatment program. Three patients had two drugs evaluated for DPT and/or RDD. Characteristics of patients and index‐IDHRs are shown in Table 1. Most patients were referred after severe or anaphylactic index‐IDHRs and/or failed re‐treatments at cancer departments. The most prevalent reaction phenotypes were *cytokine‐release (n = *27*)* and *type‐1 (n = *22*)*. Platinum salts elicited 17/22 *type‐1* reactions after a median of 9 treatments (range 6–15). In cytokine‐release reactions, the index‐IDHR occurred after a median of 2 treatments (range 1–3).

**TABLE 1:** Characteristics of patients referred to the allergy treatment program and characteristics of the index Immediate Drug Hypersensitivity Reaction (IDHR) including antineoplastic drugs involved.

Patients referred	72
Age (years): median (range)	64 (36; 83)
Gender	
Female	57
Male	15
Atopic diseases	24 (33%)
Cancer diagnosis	
Gynecologic cancer	33
Breast cancer	9
Gastrointestinal cancer	10
Malignant melanoma	4
Urologic cancer	5
Lung cancer/MUP	2
Hematologic cancer	9
Potential culprit drugs, *N* = 75	
Taxanes	23
Paclitacel	21
Docetaxel	2
Platinum salts	19
Carboplatin	12
Oxaliplatin	6
Cisplatin	1
Other chemotherapeutics	16
Liposomal doxorubicin	8
Bendamustin	5
Etoposid	1
Gemcitabin	1
D‐L‐Folinic Acid	1
Checkpoint inhibitors	12
Nivolumab	8
Ipilimumab	1
Pembrolizumab	1
Cemiplimab	1
Avelumab	1
Other biologics	5
Rituximab	3
Cetuximab	1
Bevacizumab	1
Reaction phenotype *N* = 71^a^	
Type‐1	22
Cytokine release	27
Mixed	8
Either	14
Severity of the worst index‐IDHR *N* = 71^a^	
RCUH 1	0
RCUH 2	4
RCUH 3	54
RCUH 4	13
No. of index‐IDHRs before referral *N* = 71^a^	
1	31
2	34
> 2	6

^a^ One patient was referred for a drug provocation test (DPT) without premedication in a first time treatment with paclitaxel, as the patient did not tolerate glucocorticoids. Therefore there was no index‐IDHR.

### 3.2 Skin Testing

Skin testing with 17 biologics and associated excipients was performed in 12 patients: checkpoint inhibitors (*n* = 11), *other* biologics (*n* = 6). All tests were negative.

### 3.3 Drug Provocation Tests

**FIGURE 2:**
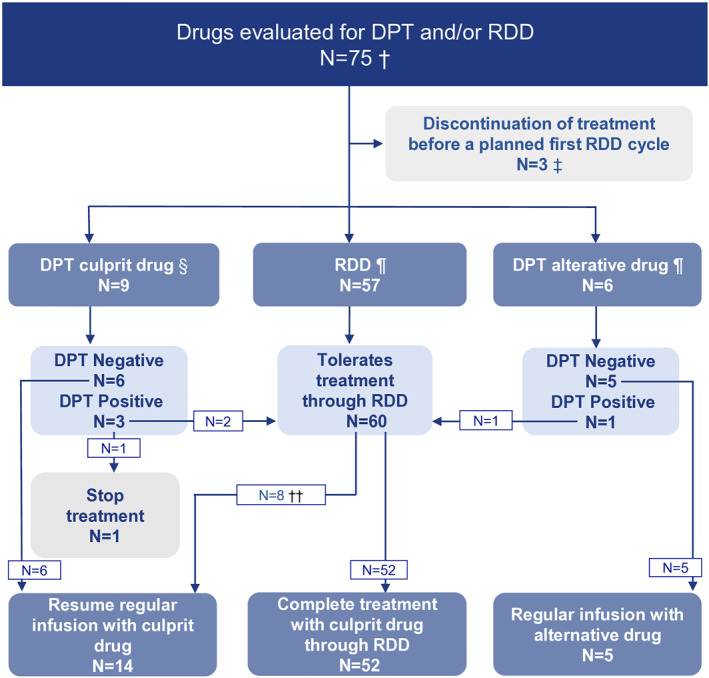
Overview of drug provocation test (DPT) and rapid drug desensitization (RDD) performed with 75 antineoplastic drugs. †Three patients had a (negative) DPT performed for one drug, whereas another drug was administered via RDD. ‡ Reasons for discontinuing treatments were: progression of cancer (*n* = 1) and complete remission of cancer (*n* = 2). § DPTs were negative for six drugs. After three positive DPTs, treatment with two drugs continued via RDD, while treatment with one drug was discontinued. ¶ DPTs were negative for five drugs and positive for one drug, which was administered via RDD in subsequent treatments. †† After uneventful RDDs, eight drugs were administered via standard infusion, which was tolerated by the patients.

Six patients underwent DPT for alternative drugs. Three had experienced life‐threatening anaphylaxis to a potentially cross‐reacting drug. DPTs were negative for five drugs. One DPT was positive for pembrolizumab (culprit: nivolumab). Pembrolizumab was administered via RDD in subsequent treatments (Figure 2).

Nine patients underwent DPT with culprit drugs. Seven based on results of the imputability analysis, and two based on a favorable risk assessment. DPTs were negative for six drugs. Of three patients with positive DPTs, two completed treatment and continued with RDD. One experienced anaphylaxis and terminated treatment (Figure 2).

Six patients underwent DPT with biologics, four of whom had a skin test. The skin test was negative in all four. Two of them subsequently had a positive DPT.

### 3.4 Rapid Drug Desensitization

#### 3.4.1 Outcome

Sixty patients received 248 RDDs, of which 247 were completed (Table 2). Eight patients tolerated standard infusion after a median of 5 RDDs (range 2–7) and resumed regular infusions in cancer departments: paclitaxel (*n* = 4), checkpoint inhibitors (*n* = 4). Of the remaining 52 patients, 35 completed their planned treatment via RDD, while 17 continued treatment until it was stopped due to side effects (*n* = 11), cancer progression (*n* = 5), or death (*n* = 1). Side effects were polyneuropathy, immunotoxicity, and palmar‐plantar erythrodysesthesia.

**TABLE 2:** Overview of patients for rapid drug desensitization (RDD) and occurrence of breakthrough reactions (BTR) in relation to drugs/drug classes.

Drug/drug class	Number of patients for RDD and number of patients with at least one BTR.				Number of RDD cycles performed and number of RDD cycles with at least one BTR.			
	The most severe BTR in each patient is indicated				The most severe BTR in a RDD cycle is indicated			
	Total	BTR	BTR	BTR	Total	BTR	BTR	BTR
		RCUH 1–2	RCUH3	RCUH4		RCUH 1‐2	RCUH 3	RCUH 4
All drugs	60	24	8	0	248^a^	59	9	0
Taxanes^b^	20	8	2	0	94	21	2	0
Platinum salts^c^	16	9	2	0	55	26	2	0
Liposomal doxorubicin	7	2	1	0	29	2	1	0
Etoposid	1	0	0	0	6	0	0	0
Bendamustine	3	1	1	0	18	1	1	0
Checkpoint inhibitors^d^	9	1	1	0	31	1	2	0
Other biologicals^e^	4	3	1	0	15^a^	8	1	0

^a^ All RDD procedures were completed except in one patient treated with rituximab. The patient resumed treatment another day and completed 6 RDD cycles.

^b^ Docetaxel: patients *n* = 1, cycles *n* = 3. Paclitaxel 80 mg/m^2^: patients *n* = 9, cycles 53. Paclitaxel 175 mg/m^2^: patients *n* = 10, cycles *n* = 38.

^c^ Carboplatin: patients *n* = 10, cycles *n* = 38. Oxaliplatin: patients *n* = 6, cycles *n* = 17.

^d^ Avelumab: patients *n* = 1, cycles *n* = 5. Cemiplimab: patients *n* = 1, cycles *n* = 3. Ipilimumab: patients *n* = 1, cycles *n* = 1. Nivolumab: patients *n* = 5, cycles *n* = 20. Pembrolizumab: patients *n* = 1, cycles *n* = 2.

^e^ Bevacizumab: patients *n* = 1, cycles *n* = 3. Cetuximab: patients *n* = 1, cycles *n* = 2. Rituximab: patients *n* = 2, completed cycles: *n* = 9.

Eleven patients underwent RDD with biologics, eight of whom had a skin test. The skin test was negative in all eight. Four of them subsequently had BTRs during the first RDD.

### 3.5 Breakthrough Reactions

BTRs occurred in 53% (32/60) of patients and in 27% (68/248) of RDD procedures. Most BTRs were mild to moderate, and no anaphylactic shocks occurred (Table 2). The proportion of BTRs was highest in RDDs involving *other* biologics and platinum salts.

**FIGURE 3:**
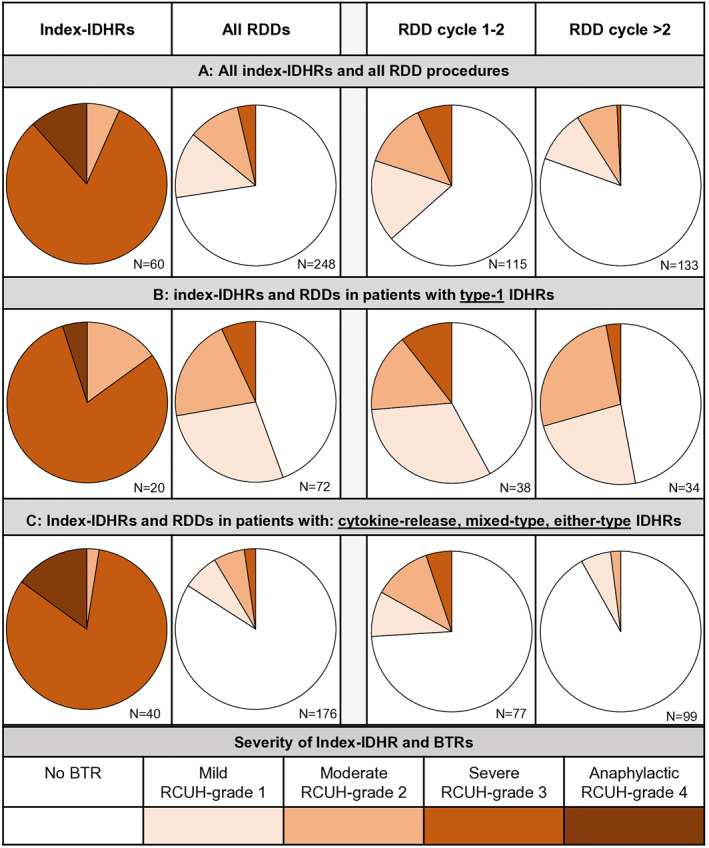
Severity of index immediate drug hypersensitivity reactions (IDHR) and fraction and severity of breakthrough reactions (BTR). The severity of index‐IDHRs is depicted alongside the fraction and severity of BTRs for all rapid drug desensitization (RDD) cycles. These are divided into phenotypes of index‐IDHR(s) and early and late RDD cycles. (A) All RDDs performed, (B) RDDs performed in patients with at least one *type‐1* phenotype IDHR (C) RDDs performed in patients with only *cytokine‐release*, *mixed‐type* or *either‐type* phenotype reactions. See Table S1 for details on the reaction phenotypes and severity grading.

The risk of BTRs was highest during first RDD and decreased in subsequent RDDs (Figure 3A). Patterns of BTRs differed between patients with type‐1 phenotype index‐IDHRs and patients with other reaction phenotypes (Figure 3B,C).

Type‐1 IDHRs were mainly observed with platinum salts. The frequency of BTRs remained high throughout successive RDDs (Figure 3B). Common symptoms included redness and itching of palms and soles, mild generalized urticaria, nasal congestion, and bronchospasm. Symptoms resolved without treatment or were controlled with antihistamines, glucocorticoids, and short‐acting beta‐agonists.

In RDD following cytokine‐release, mixed‐type or either‐type index‐IDHRs, the risk of BTRs was low, once a RDD was uneventful (Figure 3C). Common symptoms of BTRs were flushing, chest tightness and back pain. Symptoms often resolved within minutes after pausing infusion without any treatment.

A specific symptom pattern of BTRs included late‐onset symptoms of fever and/or vomiting in six patients treated with bendamustine and platinum salts. Adding glucocorticoids to premedication prevented or diminished BTRs in four patients, but was not attempted in two, where treatment was terminated due to polyneuropathy.

### 3.6 Timing

**FIGURE 4:**
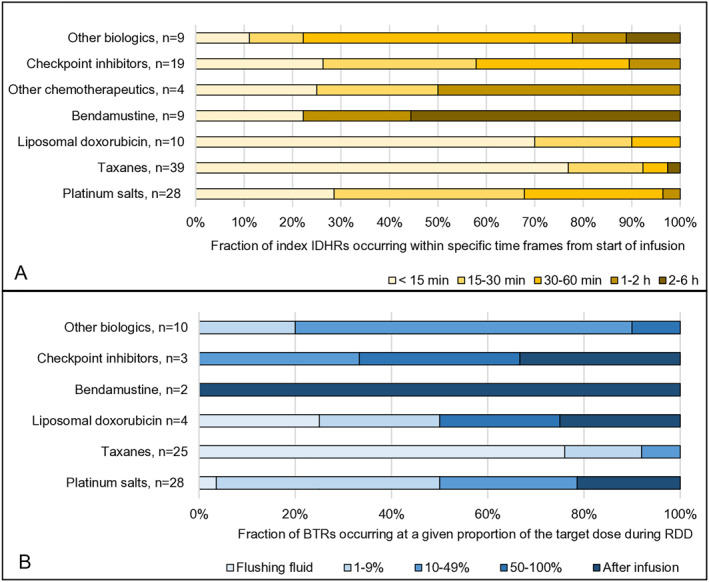
Timing of index immediate drug hypersensitivity reactions (IDHR) and breakthrough reactions (BTR) during rapid drug desensitization (RDD) in relation to drug classes. (A) The timing of index‐IDHRs is depicted in relation to drugs/drug classes. Note that some patients had more than one index‐IDHR, so the total number of index‐IDHRs is 114. (B) The timing of BTRs in RDD is depicted for different drugs/drug classes as the proportion of the target dose infused before a BTR occurred. The total number of BTRs is 72, as one patient had three BTRs and two patients had two BTRs during the same RDD treatment. Infusion sets were primed with 25–27.5 mL of flushing fluid. If a BTR occurred while patients were still receiving flushing fluid, it is indicated here as “flushing fluid”.

Timing of index‐IDHRs and BTRs varied between drug classes. With taxanes, index‐IDHRs mostly occurred within a few minutes after treatment initiation (Figure 4A). In RDDs, most BTRs occurred during infusion of the 27.5 mL of flushing fluid, and 11/25 BTRs occurred after 16–20 mL (Figure 4B). No BTRs were observed during the flush step after it was reduced to 16 mL for taxanes in January 2024. However, mild BTRs were still observed during the initial infusion steps. All severe BTRs (RCUH‐grade 3) observed in RDDs with taxanes were observed during the flush step before January 2024.

With platinum salts, the timing of index‐IDHRs and BTRs varied. Reactions occurred after both low doses and full target doses (Figure 4B). With the original one‐bag RDD‐protocol, BTRs often appeared abruptly during the final steps, where dose escalations were highest. BTRs occurred at lower doses and were less severe after reducing dose escalations in the final infusion steps in January 2024 (Table S5). See Figure 4 for information on timing of index‐IDHRs and BTRs for other drug classes.

### 3.7 Premedication and Rescue Medication

**FIGURE 5:**
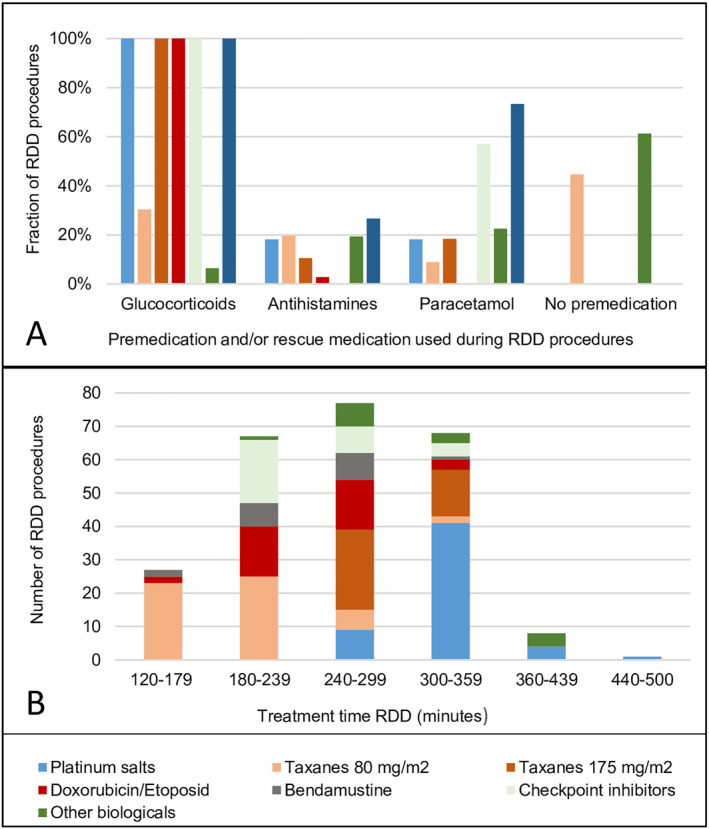
Use of premedication and rescue medication and duration of rapid drug desensitization (RDD) procedures (A) The proportion of RDD procedures involving the use of glucocorticoids, antihistamines and paracetamol are shown for different drugs/drug classes. (B) Numbers of RDD procedures completed within a specific time interval depicted for different drugs/drug classes. Treatment duration is measured from start to end of infusion, including initial and final flush step and time to treat breakthrough reactions.

Figure 5A shows the proportion of RDD‐procedures where glucocorticoids, antihistamines, and paracetamol were used as premedication and/or rescue medication. The average usage of glucocorticoids per treatment is calculated in equivalent doses of prednisolone: platinum salts: 64 mg; taxane 1‐h: 15 mg; taxane 3‐h: 71 mg; liposomal doxorubicin/etoposide: 51 mg; checkpoint inhibitors: 3 mg; *other* biologics: 77 mg; and bendamustine: 58 mg. Antihistamines were only used to treat BTRs. Glucocorticoids were only used in combination with checkpoint inhibitors as standard premedication to prevent nausea when treatments included platinum salts. Omalizumab was used in three patients. One quadrupled the dose eliciting a BTR. One had no type‐1 symptoms during RDD, but developed fever and vomiting hours after discharge. One experienced no further BTRs.

### 3.8 Treatment Duration

Figure 5B shows the number of RDD procedures completed within specific time intervals. Treatment duration was below 6 hours in 239/248 (96%) RDD procedures and ranged from 2.5 to 7.5 h.

Of 248 antineoplastic treatments involving RDD, 227 were completed in one day at the Allergy Center, while 21 involved the administration of additional drugs at the cancer department on another day. Of 248 treatments performed at the Allergy Center, 158 included one antineoplastic drug, 70 included two drugs, 19 included three drugs, and one included four drugs. Nineteen treatments were completed in three hours, 49 in four hours, 53 in five hours, 90 in six hours, 34 in seven hours, and three in eight hours.

## 4 Discussion

During 28 months, 75 drugs were evaluated using DPT and/or RDD in 72 unique patients. Through DPT, a safe drug alternative was found for five drugs, hypersensitivity was ruled out for six, and one treatment was terminated. RDD was indicated in 63 patients, and 60 patients reached a first treatment. Of 248 initiated RDD procedures, 247 were completed. BTRs were observed in 53% of patients and 27% of RDD procedures. Most BTRs were mild to moderate. The treatment duration was below 6 hours in 96% of RDD procedures.

The one‐bag RDD‐protocol used in the study was efficient, since all patients were able to continue treatment consistent with previously published results [2]. The proportion of BTRs in RDD procedures was within the generally reported limit [2], and no anaphylactic reactions occurred, unlike in other studies [6, 8, 12, 15, 18, 25, 30]. The proportion of patients with BTRs was higher than generally reported [2], but comparable to three specific studies [18, 19, 30]. Discordance may be explained by differences in study population. Patients with mild to moderate single index‐IDHRs were generally not referred for RDD in this study.

BTRs were frequent in RDDs if *type‐1* symptoms were present during index‐IDHRs, which was often the case with platinum salts. Tailored infusion plans with reduced dose escalations in the final steps did not prevent BTRs, but BTRs occurred at lower doses and were milder. BTRs rarely recurred during the same RDD procedure, once symptoms had resolved [3]. This phenomenon may be explained by *the empty mast cell syndrome,* in which mast cells undergo a period of unresponsiveness following activation [2, 31]. Considering this, it seems reasonable to accept mild BTRs in RDDs involving platinum salts/*type‐1* phenotype reactions. Avoiding antihistamines in premedication can help identify BTRs, while symptoms are still mild, which may prevent more severe BTRs from developing. Omalizumab effectively prevented BTRs in one patient and can be useful in selected cases [2].

Recording timing of BTRs allowed for rational tailoring of standard infusion plans for individual drug classes. The flush step should be drug‐free or contain such a small drug dose that patients do not react to it. In this study, the volume of the flush step was adapted in January 2024 to the minimum volume infused before BTRs were observed. The optimal volume to be infused during the flush step depends on specific drugs and infusion set used.

Adaptions of RDD‐protocols led to shorter treatment duration for some RDD procedures. This was particularly relevant for taxanes and checkpoint inhibitors. Other studies have gradually decreased infusion duration after uneventful RDDs with paclitaxel [7, 32], but this has not been standardized for other drugs. Using multiple infusion plans for different drug classes was possible, since the high‐precision infusion pump allowed an unlimited number of encoded programs.

Treatment duration is important to both patients and institutions. However, only two studies report the average duration of RDD procedures [19, 22]. Our results show that most antineoplastic treatments involving RDD can be carried out at an allergy center during daytime clinic hours and completed within one day.

### 4.1 Strength and Limitations

This is the first one‐bag RDD‐protocol where infusion sets are primed with flushing fluid, as priming with drug solution is prohibited according to Danish legislation. It is unpredictable when the first drug molecule reaches the patient. Therefore, it was important to record the timing of BTRs in order to determine the optimal volume to infuse during the flush step. Culprit drugs were not administered in precise doses during the initial infusion steps. During these steps, a mixture of flushing fluid and drug solution was infused, and patients went from receiving highly diluted drug concentrations to full drug concentrations.

Skin testing was not performed with chemotherapeutics, and biomarkers were not measured in cancer departments or during BTRs. One limitation is that these diagnostic tests were omitted. However, the diagnostic value of skin testing is not well validated [3, 33]. Low sensitivity has been reported [11, 25, 34], as it may be affected if performed in non‐IgE‐mediated reactions or too early following IDHRs [2, 9]. Skin testing is used for risk stratification. The results of this study suggest that phenotyping of IDHRs can be used to identify RDDs with high risk of BTRs. Phenotyping is cost‐free, while skin testing with antineoplastic drugs and in vitro diagnostics are expensive and carry risks to staff and environment.

### 4.2 Conclusion

This study is the first of its kind in Northern Europe and the first to use a one‐bag RDD‐protocol with drug concentrations strictly following manufacturer's recommendations and infusion sets primed with flushing fluid. The study contributes with previously unpublished detailed information on timing of BTRs, total use of premedication and rescue medication, and the actual treatment duration of RDD procedures. The protocol ensured that patients received the planned courses of treatment with minimal side effects.

## Author Contributions

T.H.R. and C.B.J. conceived the study design. T.H.R., L.K.T., M.N.B. and H.M.R. evaluated the patients. D.G.M. and T.H.R. developed a drug solution delivery system for rapid drug desensitization. All authors were involved in data interpretation. T.H.R. wrote the manuscript. All authors reviewed and approved the final manuscript.

## Conflicts of Interest

The authors declare no conflicts of interest.

## Supporting information


**Table S1** The RCUH‐classification [12] of drug hypersensitivity to chemotherapeutics and biological agents and a model for phenotyping immediate drug hypersensitivity reactions [25].

Supporting Information S1

Supporting Information S2

**Table S4** Standard infusion plans for standard infusion bag volumes

**Table S5** Adjusted standard one‐bag RDD‐protocols for taxane 1‐hour, taxane 3‐hour, platinum salts and checkpoint inhibitors, here depicted for the most commonly used volumes in the infusion bags

## Data Availability

The data that support the findings of this study are available on request from the corresponding author. The data are not publicly available due to privacy or ethical restrictions.
