# Supplementary material for: Implementation of Rapid Drug Desensitization in Antineoplastic Drug Therapy in Denmark Using One‐Bag Protocols
Source: Clin Transl Allergy. 2025 Aug 13;15(8):e70093. doi: 10.1002/clt2.70093 (PMC12350077; doi:10.1002/clt2.70093)
Supplement: Supplementary file 1 — Table S1 The RCUH‐classification [12] of drug hypersensitivity to chemotherapeutics and biological agents and a model for phenotyping immediate drug hypersensitivity reactions [25]. [file CLT2-15-e70093-s003.pdf]

Supplementary 1: The RCUH-classification [12] of drug hypersensitivity to chemotherapeutics and biological agents and a model for phenotyping immediate drug hypersensitivity reactions [25]

The RCUH Severity grading system [12]

	Time to onset of the DHR	Symptoms suggestive of mast cell-mediated reactions	Symptoms suggestive of cytokine release	Common symptoms to both reaction types
**Grade 1: Mild reaction**	Not defined	Pruritus, local urticarial or angioedema	Fever/chills (<38°C), mild Back pain	Erythema
**Grade 2: Moderate reaction**<br>*If a grade 2 symptom is present, any grade 1 symptoms are included*	Slow onset >15 min	Generalized urticarial and/or angioedema, coryzal symptoms, irritative cough, throat tightness	Severe back pain, chest pain, fever (>38°C)	Dyspnea (SpO_2>92%) nausea, abdominal pain
**Grade 3: Severe reaction**<br>*If there is rapid onset of a grade 2 symptom, it is defined as a grade 3 reaction.*<br><br>*If a grade 3 symptom is present, any grade 1-2 symptoms are included*	Rapid onset <15 min of grade 2 symptoms<br><br>or<br><br>a grade 3 symptom at a not defined time point	Grade 2 symptoms<br><br>Throat tightness combined with dysphagia, dysphonia or stridor, wheezing	Grade 2 symptoms	Grade 2 symptoms<br><br>Chest tightness, vomiting, diarrhea, dyspnea (SpO_2<92%), diaphoresis, dizziness, hypertension
**Grade 4: Anaphylactic shock**<br>*If a grade 4 symptom is present, any grade 1-3 symptoms are included*	Immediate onset or rapid progression			Hypotension, cyanosis, sense of impending doom, faintness, loss of sphincter control, cardiovascular or respiratory arrest

Model for phenotyping Immediate drug hypersensitivity reaction [25]

	At least one of these symptom must be present	None of these symptoms must be present	These unspecific symptoms may be included in all reaction phenotypes
**Type-1**	Pruritus, urticaria, angioedema, nasal congestion, sneezing, wheezing, cough, throat tightness and tongue swelling	Cytokine-release symptoms	Flushing, warmth, erythema, unspecific rashes<br><br>Dyspnea, oxygen desaturation, chest tightness<br><br>Tachycardia, presyncope, syncope, hypertension, hypotension<br><br>Nausea, vomiting, abdominal pain, diarrhea, bloating, reflux<br><br>Numbness, weakness, seizures, unusual taste, diaphoresis
**Cytokine-release**	Chest pain, back pain, headache, rigor, other pain, chills and fever	Type-1 symptoms	
**Mixed-type**	At least one type-1 symptom<br><br>and<br><br>At least one cytokine-release symptoms		
**Either-type**	Unspecific symptoms	Type-1 symptoms and Cytokine-release symptoms	
