# Supplementary material for: Implementation of Rapid Drug Desensitization in Antineoplastic Drug Therapy in Denmark Using One‐Bag Protocols
Source: Clin Transl Allergy. 2025 Aug 13;15(8):e70093. doi: 10.1002/clt2.70093 (PMC12350077; doi:10.1002/clt2.70093)
Supplement: Supplementary file 2 — Supporting Information S1 [file CLT2-15-e70093-s002.pdf]

Supplementary 2: Algorithm for managing patients referred to the allergy treatment program

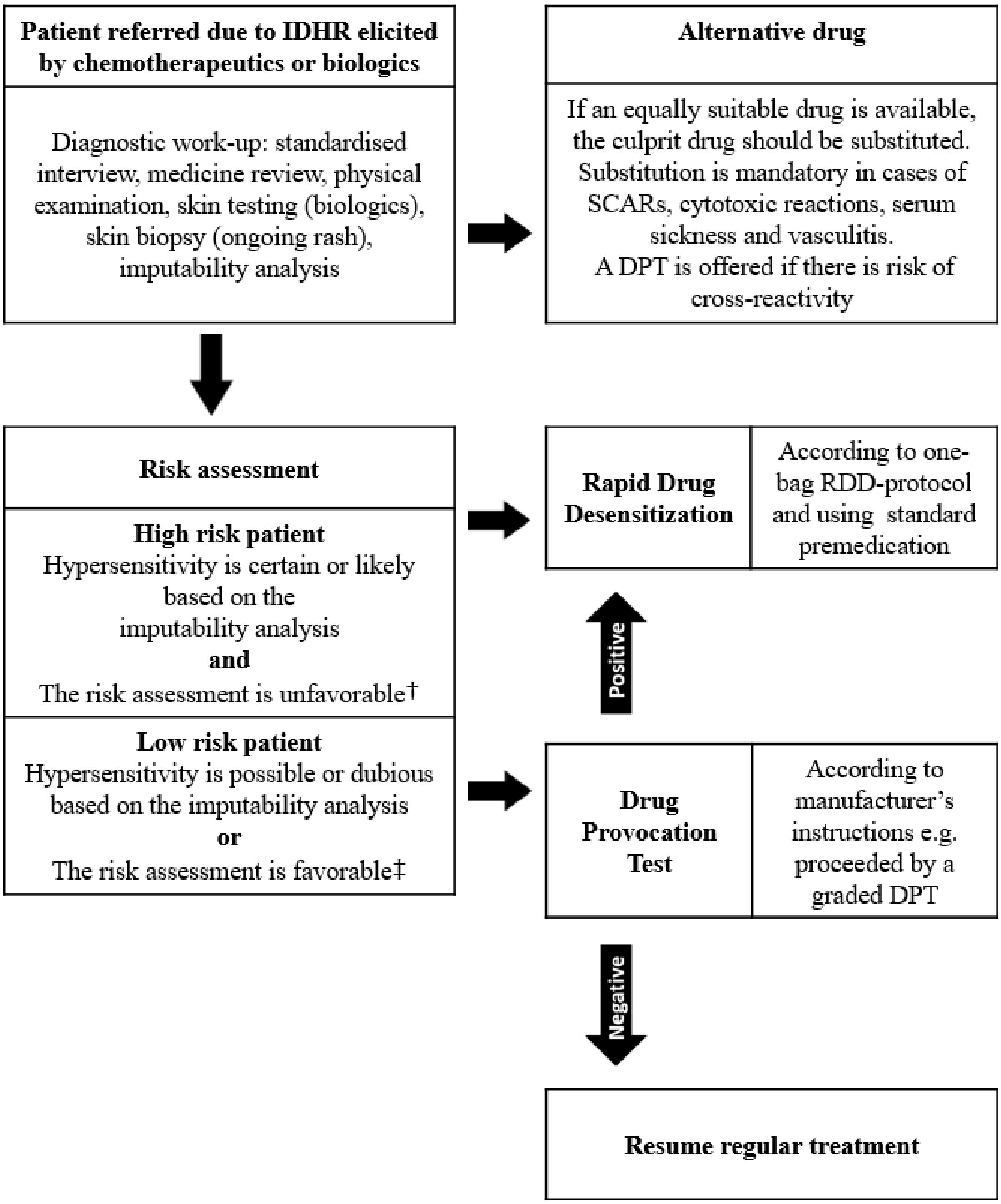

† Unfavourable risk assessment includes RCUH-grade 4 reaction **or** a type-1 phenotype reaction **or** a repeated reaction during re-treatment at cancer departments involving a decreased infusion rate ≤ 50% of a standard infusion rate.

‡ Favourable risk assessment includes mild to severe RCUH-grade 1-3 reactions **and** one of the reaction phenotypes: Cytokine-release mixed-type or either-type

Other factors to include in the risk assessment are: Reactions to drugs previously tolerated by standard infusion, mastocytosis, significant cardiovascular disease or lung disease, acute critical illness such as infection and fragile patient: performance status 3-4.

Abbreviations: DPT: drug provocation test, IDHR: immediate drug hypersensitivity reaction, RDD: rapid drug desensitization, SCAR: severe cutaneous adverse reactions
