# Supplementary material for: Implementation of Rapid Drug Desensitization in Antineoplastic Drug Therapy in Denmark Using One‐Bag Protocols
Source: Clin Transl Allergy. 2025 Aug 13;15(8):e70093. doi: 10.1002/clt2.70093 (PMC12350077; doi:10.1002/clt2.70093)
Supplement: Supplementary file 3 — Supporting Information S2 [file CLT2-15-e70093-s004.pdf]

Supplementary 3:

Overview of drug solutions used for rapid drug desensitization: For selected drugs, alphabetically

Avelumab
One standard dosage: 800 mg
The manufacturer recommends a 250 mL solution
Volume of infusion bags:

- 265 mL NaCl

Flushing fluid: NaCl, 9 mg/ml
Filter: Yes

Bendamustine
Standard dosages of 70-120 mg/m^2 are used
The manufacturer recommends a 500 mL solution
Volumes of infusion bags:

- 50 mg of bendamustine is delivered in 525 mL NaCl at 9.15 am, the rest of the target dose is delivered in 525 mL NaCl at 11.15 am.

Flushing fluid: NaCl, 9 mg/ml
Filter: No
Special attention: Shelf life: 3.5 hours

Bevacizumab
Four standard dosages are used: 5 mg/kg, 7.5 mg/kg, 10 mg/kg and 15 mg/kg.
The manufacturer states a concentration range of 1.4-16.5 mg/mL
Volume of infusion bags:

- 110 mL NaCl

Flushing fluid: NaCl, 9 mg/ml
Filter: No

Carboplatin
Four standard dosages are used: AUC 1.5, AUC 4, AUC 5 and AUC 6. (dosage depends on kidney function)
The manufacturer states a minimum concentration of 0.5 mg/mL
Volumes of infusion bags:

- 110 mL glucose in dosages between: 55-132.49 mg
- 265 mL glucose in dosages between: 132.5-262.49 mg
- 525 mL glucose in dosages at 262.5 mg and above

Flushing fluid: NaCl, 9 mg/ml
Filter: No

Cemiplimab
One standard dosage: 350 mg
The manufacturer states a concentration range of 1-20 mg/mL
Volume of infusion bags:

- 265 mL

Flushing fluid: NaCl, 9 mg/ml
Filter: Yes

Cetuximab
Four standard dosages are used: 250 mg/m^2, 400 mg/m^2 and 500 mg/m^2
The manufacturer states no requirements for the concentration of the drug solution
Volume of infusion bags:

- 525 mL NaCl

Flushing fluid: NaCl, 9 mg/ml
Filter: No

Docetaxel
Four standard dosages are used: 40 mg/m^2, 50 mg/m^2, 75 mg/m^2 and 100 mg/m^2
The manufacturer states a concentration range of 0.3-0.74 mg/mL
Volumes of infusion bags:

- 110 mL NaCl in dosages between: 33-79.99 mg
- 265 mL NaCl in dosages between: 80-195.99 mg
- 525 mL NaCl in dosages between: 196-387.99 mg

Flushing fluid: NaCl, 9 mg/ml
Filter: No

Doxorubicin, pegylated liposomal
Two standard dosages are used: 30 mg/m^2 and 40 mg/m^2
The manufacturer recommends a 250 mL solution
Volume of infusion bags:

- 265 mL glucose

Flushing fluid: Glucose, 50 mg/mL
Filter: No

Etoposid
Three standard dosages are used: 100 mg/m^2, 120 mg/m^2 and 200 mg/ m^2
The manufacturer states a concentration range of 0.2-0.4 mg/mL
Volumes of infusion bags:

- 265 mL NaCl in dosages between: 53-105.99 mg
- 525 mL NaCl in dosages between: 106-209.99 mg
- 1025 mL NaCl in dosages between of 210-410 mg

Flushing fluid: NaCl, 9 mg/mL
Filter: No

Ipilimumab
Two standard dosages are used: 1 mg/kg and 3 mg/kg
The manufacturer states a concentration range of 1-4 mg/mL
Volumes of infusion bags:

- 60 mL NaCl in dosages between 60-109.99 mg
- 110 mL NaCl in dosages between 110-264.99 mg
- 265 mL NaCl in dosages of 265 and above

Flushing fluid: NaCl, 9 mg/mL
Filter: Yes

Nivolumab
Three standard dosages are used: 3 mg/kg, 4.5 mg/kg and 6 mg/kg (up to 480 mg)
The manufacturer states a concentration range of 1-10 mg/mL
Volumes of infusion bags:

- 60 mL NaCl in dosages between 60-109.99 mg
- 110 mL NaCl in dosages between 110-264.99 mg
- 265 mL NaCl in dosages of 265 and above

Flushing fluid: NaCl, 9 mg/mL
Filter: Yes

Oxaliplatin (Accord)
Three standard dosages are used: 60 mg/m^2, 85 mg/m^2 and 130 mg/m^2
The manufacturer states a concentration range of 0.2-0.7 mg/mL
Volumes of infusion bags:

- 110 mL Glucose in dosages between: 22-52.99 mg
- 265 mL Glucose in dosages between: 53-104.99 mg
- 525 mL Glucose in dosages between: 105-367 mg

Flushing fluid: Glucose, 50 mg/mL
Filter: No

Paclitaxel
Three standard dosages are used: 80 mg/m^2, 175 mg/m^2 and 250 mg/m^2.
The manufacturer states a concentration range of 0.3-1.2 mg/mL
Volumes of infusion bags:

- 110 mL NaCl in dosages between: 33-79.99 mg.
- 265 mL NaCl in dosages between: 80-209.99 mg
- 525 mL NaCl in dosages between: 210-599.99 mg
- 1025 mL NaCl in dosages between: 600-1230 mg

Flushing fluid: NaCl, 9 mg/mL
Filter: Yes

Pembrolizumab
Four standard dosages are used: 2 mg/kg, 4 mg/kg, 200 mg and 400 mg
The manufacturer states a concentration range of 1-10 mg/mL
Volumes of infusion bags:

- 60 mL NaCl in dosages between 60-109.99 mg
- 110 mL NaCl in dosages between 110-264.99 mg
- 265 mL NaCl in dosages of 265 and above

Flushing fluid: NaCl, 9 mg/mL
Filter: Yes

Rituximab (Rixathon sandoz)
Four standard dosages are used: 375 mg/m^2, 500 mg/m^2, 500 mg and 1000 mg
The manufacturer states a concentration range of 1-4 mg/mL
Volumes of infusion bags:

- 265 mL NaCl in dosages of 265-524.99 mg
- 525 mL NaCl in dosages of 525 mg and above

Flushing fluid: NaCl, 9 mg/mL
Filter: No
