# Supplementary material for: Implementation of Rapid Drug Desensitization in Antineoplastic Drug Therapy in Denmark Using One‐Bag Protocols
Source: Clin Transl Allergy. 2025 Aug 13;15(8):e70093. doi: 10.1002/clt2.70093 (PMC12350077; doi:10.1002/clt2.70093)
Supplement: Supplementary file 4 — Table S4 Standard infusion plans for standard infusion bag volumes [file CLT2-15-e70093-s001.pdf]

Supplementary 4: Standard infusion plans for standard infusion bag volumes

	Percentage of a normal infusion rate	Percentage of target dose each step	Cumulative percentage of target dose	Infusion rate ml/h (volume each step/infusion time in minutes each step)				
				Volume 60 (85ml†)	Volume 110 (135ml†)	Volume 265 (290ml†)	Volume 525 (550ml†)	Volume 1025 (1050ml†)
Flush step				80 (20/15)	80 (20/15)	80 (20/15)	80 (20/15)	80 (20/15)
Step 1	0.13	0.03	0.03	0.08 (0.02/15)	0.12 (0.03/15)	0.4 (0.1/15)	0.6 (0.2/15)	1.2 (0.3/15)
Step 2	0.25	0.06	0.09	0.16 (0.04/15)	0.28 (0.07/15)	0.8 (0.2/15)	1.2 (0.3/15)	2.4 (0.6/15)
Step 3	0.5	0.13	0.22	0.4 0.1/15)	0.4 0.1/15)	1.2 (0.3/15)	2.8 (0.7/15)	5.2 (1.3/15)
Step 4	1	0.3	0.5	0.8 (0.2/15)	1.2 (0.3/15)	2.8 (0.7/15)	5.2 (1.3/15)	10.4 (2.6/15)
Step 5	2	0.5	1	1.2 (0.3/15)	2.4 (0.6/15)	5.2 (1.3/15)	10.4 (2.6/15)	20.4 (5.1/15)
Step 6	4	1	2	2.4 (0.6/15)	4.4 (1.1/15)	10.8 (2.7/15)	21.2 (5.3/15)	41.2 (10.3/15)
Step 7	8	2	4	4.8 (1.2/15)	8.8 (2.2/15)	21.2 (5.3/15)	42 (10.5/15)	82 (20.5/15)
Step 8	16	4	8	9.6 (2.4/15)	17.6 (4.4/15)	42.4 (10.6/15)	84 (21/15)	164 (41/15)
Step 9	32	8	16	19.2 (4.8/15)	35.2 (8.8/15)	84.8 (21.2/15)	168 (42/15)	328 (82/15)
Step 10	50	84	100	29.9 (55.4/111)	55.1 (97.4/106)	132.6 (227.6/103)	262.4 (446.1/102)	514.6 (866.3/101)
Final flushing				300 (75/15)	300 (75/15)	300 (75/15)	300 (75/15)	300 (75/15)
Total infusion time				4 h and 36 min	4h and 31 min	4h and 28 min	4h and 27 min	4h and 26 min

† The volume of the flushing fluid is included in the infusion plan. The volume contained in the infusion set is 25 ml. The total volume (flushing fluid + drug solution) is infused in the flush step and step 1-10, before increasing the infusion rate in the final flush step. A program for infusion of the flush step and steps 1-10 is coded in high precision infusion pumps (Infusomat Space ® Braun).
