# Supplementary material for: Implementation of Rapid Drug Desensitization in Antineoplastic Drug Therapy in Denmark Using One‐Bag Protocols
Source: Clin Transl Allergy. 2025 Aug 13;15(8):e70093. doi: 10.1002/clt2.70093 (PMC12350077; doi:10.1002/clt2.70093)
Supplement: Supplementary file 5 — Table S5 Adjusted standard one‐bag RDD‐protocols for taxane 1‐hour, taxane 3‐hour, platinum salts and checkpoint inhibitors, here depicted for the most commonly used volumes in the infusion bags [file CLT2-15-e70093-s005.pdf]

Supplementary 5: Adjusted standard one-bag RDD-protocols for taxane 1-hour, taxane 3-hour, platinum salts and checkpoint inhibitors, here depicted for the most commonly used volumes in the infusion bags

	Taxane – Normal infusion time: 1 hour<br><br>Paclitaxel 80mg/m2/Docetaxel<br><br>Infusion bag 265 ml<br>Infusion set + filter: 27.5.ml<br>Total volume: 292.5ml†				Taxane – Normal infusion time: 3 hours<br><br>Paclitaxel 175mg/m2<br><br>Infusion bag 525ml<br>Infusion set + filter: 27.5ml<br>Total volume: 552.5ml†			
	Percentage of normal infusion rate	Percentage of target dose each step	Cumulative percentage of target dose	Infusion rate ml/h<br><br>(volume each step/infusion time in minutes each step)	Percentage of normal infusion rate	Percentage of target dose each step	Cumulative percentage of target dose	Infusion rate ml/h<br><br>(volume each step/infusion time in minutes each step)
Flush Step				64<br>(16/15)				64<br>(16/15)
Step 1	1	0.3	0.3	2.8<br>(0.7/15)	1	0.1	0.1	1.6<br>(0.4/15)
Step 2	2	0.5	0.8	5.2<br>(1.3/15)	2	0.2	0.3	3.6<br>(0.9/15)
Step 3	4	1.0	1.8	10.8<br>(2.7/15)	4	0.3	0.6	7.2<br>(1.8/15)
Step 4	8	2.0	3.8	21.2<br>(5.3/15)	8	0.7	1.3	14<br>(3.5/15)
Step 5	16	4.0	7.8	42.4<br>(10.6/15)	16	1.3	2.6	28<br>(7/15)
Step 6	24	6.0	13.8	63.6<br>(15.9/15)	24	2.0	4.6	42<br>(10.5/15)
Step 7	36	9.0	22.8	95.6<br>(23.9/15)	36	3.0	7.6	63.2<br>(15.8/15)
Step 8	50	12.5	35.6	132.4<br>(33.1/15)	50	4.2	11.8	87.6<br>(21.9/15)
Step 9	67	16.8	52	176.8<br>(44.2/15)	67	5.6	17.3	117.2<br>(29.3/15)
Step 10	83	20.8	72.8	220<br>(55/15)	83	6.9	24.3	145.6<br>(36.4/15)
Step 11	100	27.3	100	265<br>(83.8/19)	100	75.8	100	175<br>(409/140)
Final flushing				300<br>(75/15)				300<br>(75/15)
Total infusion time				3 h and 19 min				5 h and 20 min

† The volume of the flushing fluid is included in the infusion plan. The volume of the infusion set (25 ml) + the volume contained in an in-line filter (2.5ml). The total volume (flushing fluid + drug solution) is infused during the flush step and steps 1-11 before increasing the infusion rate in the final flush step. A program for the flush step and infusion steps 1-11 is coded in high precision infusion pumps (Infusomat Space® Braun).

Supplementary 5: Continued

	Platinum salts – Normal infusion time: 1 hour<br>Carboplatin/oxaliplatin/cisplatin<br><br>Infusion bag 525 ml‡<br>Infusion set: 25 ml<br>Total volume: 550ml§				Checkpoint inhibitors – Normal infusion time: 1 hour<br>e.g. Nivolumab, Avelumab, Pembrolizumab<br><br>Infusion bag 265ml‡<br>Infusion set + filter: 27.5ml<br>Total volume: 292.5ml§			
	Percentage of a normal infusion rate	Percentage of target dose each step	Cumulative percentage of target dose	Infusion rate ml/h<br><br>(volume each step/infusion time in minutes each step)	Percentage of a normal infusion rate	Percentage of target dose each step	Cumulative percentage of target dose	Infusion rate ml/h<br><br>(volume each step/infusion time in minutes each step)
Flush step				80<br>(20/15)				80<br>(20/15)
Step 1	0.13	0.03	0.03	0.8<br>(0.2/15)	1	0.3	0.3	2.8<br>(0.7/15)
Step 2	0.25	0.06	0.09	1.2<br>(0.3/15)	2	0.5	0.8	5.2<br>(1.3/15)
Step 3	0.5	0.13	0.22	2.8<br>(0.7/15)	4	1	1.8	10.8<br>(2.7/15)
Step 4	1	0.3	0.5	5.2<br>(1.3/15)	8	2	3.8	21.2<br>(5.3/15)
Step 5	2	0.5	1.0	10.4<br>(2.6/15)	12	3	6.8	32<br>(8/15)
Step 6	4	1	2.0	21.2<br>(5.3/15)	16	4	10.8	42.4<br>(10.6/15)
Step 7	8	4	6.0	42<br>(21/30)	20	10	20.8	53<br>(26.5/30)
Step 8	16	8	14.0	84<br>(42/30)	24	12	32.8	63.6<br>(31.8/30)
Step 9	24	12	26.0	126<br>(63/30)	28	14	46.8	74.2<br>(37.1/30)
Step 10	32	16	42.0	168<br>(84/30)	30	15	61.8	79.4<br>(39.7/30)
Step 11	40	58	100.0	211<br>(309.6/88)	32	38.3	100	84.8<br>(108,8/77)
Final flushing				300<br>(75/15)				300<br>(75/15)
Total infusion time				5 h and 29 min				5 h and 17 min

‡ In these examples, the volume of the infusion bags are 525 ml and 265 ml for platinum salts and checkpoint inhibitors, respectively. The volume will differ depending on the target dose in order to comply with manufacturer's recommendations on drug concentrations.

§ The volume of the flushing fluid is included in the infusion plan. The volume of the infusion set (25 ml) + the volume contained in an in-line filter (2.5ml) for checkpoint inhibitors. The total volume (flushing fluid + drug solution) is infused during the flush step and steps 1-11 before increasing the infusion rate in the final flush step. A program for the flush step and infusion steps 1-11 is coded on high precision infusion pumps (Infusomat Space® Braun).
